# Effects of linalyl acetate on oxidative stress, inflammation and endothelial dysfunction: can linalyl acetate prevent mild cognitive impairment?

**DOI:** 10.3389/fphar.2023.1233977

**Published:** 2023-07-28

**Authors:** You Kyoung Shin, Geun Hee Seol

**Affiliations:** ^1^ Department of Basic Nursing Science, College of Nursing, Korea University, Seoul, Republic of Korea; ^2^ BK21 FOUR Program of Transdisciplinary Major in Learning Health Systems, Graduate School, Korea University, Seoul, Republic of Korea

**Keywords:** linalyl acetate, prevention, oxidative stress, inflammation, endothelial dysfunction, mild cognitive impairment

## Abstract

Mild cognitive impairment (MCI) is a major public health challenge with an increasing prevalence. Although the mechanisms underlying the development of MCI remain unclear, MCI has been reported to be associated with oxidative stress, inflammatory responses, and endothelial dysfunction, suggesting that agents that reduce these factors may be key to preventing MCI. Currently, no agents have been approved for the treatment of MCI, with the efficacy of commonly prescribed cholinesterase inhibitors remaining unclear. Relatively safe natural products that can prevent the development of MCI are of great interest. Linalyl acetate (LA), the major component of clary sage and lavender essential oils, has been shown to have a variety of pharmacological effects, including anti-hypertensive, anti-diabetic, neuroprotective, anti-inflammatory, and antioxidant properties, which may have the potential for the prevention of MCI. The present review briefly summarizes the pathogenesis of MCI related to oxidative stress, inflammatory responses, and endothelial dysfunction as well as the benefits of LA against these MCI-associated factors. The PubMed and Google Scholar databases were used to search the relevant literature. Further clinical research may lead to the development of new strategies for preventing MCI, particularly in high-risk populations with oxidative stress, inflammatory responses, and endothelial dysfunction (e.g., patients with hypertension and/or diabetes mellitus).

## 1 Introduction

Mild cognitive impairment (MCI) is a condition in which individual experiences a moderate cognitive decline greater than that expected during normal aging (Anderson, 2019). The global prevalence of MCI in community-dwelling adults has been estimated to be about 15.6% (Bai et al., 2022). The number of people living with MCI in 2020 in the United States was 12.2 million, which has been estimated to increase to 21.6 million by 2060 (Rajan et al., 2021). Individuals with MCI have a poorer quality of life and a greater cost burden than healthy individuals (Tahami Monfared et al., 2022), with a large proportion of caregivers of individuals with MCI reporting caregiver burden (Connors et al., 2019). Moreover, approximately one-third of people with MCI progress to dementia (Glynn et al., 2021). Preventing the development of MCI may therefore prevent possible progression to dementia in aging populations.

Although the exact mechanisms underlying the development of MCI remain unclear, oxidative stress is frequently observed in MCI patients. Modifications of protein structures (e.g., α-enolase, glucose-regulated protein precursor) induced by oxidative stress were found to be more frequent in MCI brains than in control brains (Butterfield, 2023), and oxidative stress and nitrosative stress/antioxidant ratio were reported to be higher in individuals with MCI than in healthy controls (Nantachai et al., 2022). MCI patients showed significantly increased serum malondialdehyde (MDA) levels compared with age-matched healthy controls (Padurariu et al., 2010). In addition, nicotinamide adenine dinucleotide phosphate oxidase (NOX) 2 expression in the brain vascular fraction was significantly elevated in patients with vascular cognitive impairment compared to control cases suggesting vascular contribution to cognitive decline (Alfieri et al., 2022). In addition, oxidative stress was found to be the mechanistic link between age-related disorders and MCI. For example, hypertension-induced reactive oxygen species (ROS) cause oxidative damage to cerebral endothelial cells, resulting in blood-brain barrier (BBB) disruption (Ungvari et al., 2021). Moreover, in patients with the common age-related metabolic disorder diabetes mellitus (Natarajan et al., 2020), peripheral metabolic alterations increase oxidative stress and neuro-inflammation, which negatively affect cognitive function (Dutta et al., 2022).

Although several studies have reported that oxidative stress plays an important role in MCI, clinical studies of antioxidants in MCI patients have yielded inconsistent results (Alavi Naeini et al., 2014; Rita Cardoso et al., 2016). The discrepancies among these studies may stem from the multifactorial characteristics of MCI. For example, one study found that brachial flow-mediated dilatation was significantly related to MCI in an elderly population, suggesting a pivotal role of endothelial dysfunction in MCI (Vendemiale et al., 2013). Another study, however, found that serum inflammatory markers such as interleukin (IL)-6 and high-sensitivity C-reactive protein levels were associated with the risk of MCI in patients with type 2 diabetes mellitus (Zheng et al., 2019). And a third study reported that MCI patients with high plasma matrix metalloproteinase (MMP)-9 levels show a faster rate of cognitive decline (Abe et al., 2020). Another consideration when exploring the factors giving rise to MCI is that oxidative stress, inflammation, and endothelial dysfunction are closely linked (Higashi, 2022). Given the multifactorial nature of MCI, in this review we explore the notion that reducing oxidative stress, inflammation, and endothelial dysfunction, not just oxidative stress, may be key to preventing MCI.

To date, no drugs have been found that cure MCI (Alvi et al., 2022). Cholinesterase inhibitors are commonly used to delay progression of MCI or to improve cognition in MCI patients, but their efficacy remains inconclusive. For example, although donepezil showed limited efficacy in improving the cognitive function of individuals with MCI, it could not delay MCI progression (Zhang et al., 2022). Moreover, donepezil was associated with significantly higher rates of adverse effects, such as diarrhea and vomiting, than placebo (Zhang et al., 2022). Efforts are underway to develop complementary and integrative therapeutic regimens using natural products for cognitive disorders as such products are perceived to constitute a safer and more natural option than conventional medicine (Nguyen et al., 2022). For instance, mangiferin, a natural glucoxilxanthone, has been found to show protective effects against memory impairment in animals and humans, without any side effects at the selected doses (Lum et al., 2021). Also, previous studies have suggested that the naturally occurring compounds genistein and celastrol are promising molecules for the development of neuroprotective drugs (Fuloria et al., 2022; Amir Yusri et al., 2023).

Linalyl acetate (3,7-dimethyl-1,6-octadien-3-yl acetate; LA) is a major volatile component of the essential oils of *Salvia sclarea* (clary sage) and *Lavandula angustifolia* (lavender) (Seol et al., 2013). LA is used as a fragrance ingredient in shampoos, detergents, and cosmetic products (Letizia et al., 2003). Humans are exposed to LA not only through fragrances but also through the consumption of flavored teas such as Earl Grey tea, a cup of which contains 0.2 mg of LA (Orth et al., 2014). Repeated-dose and reproductive toxicity studies showed that exposure to 36 mg/kg/day and 200 mg/kg/day LA, respectively, did not have any adverse effects (Api et al., 2015). Computational analyses showed that LA was neither a substrate nor an inhibitor of cytochromes involved in the metabolism of neuropsychiatric drugs (Avram et al., 2021). In addition, the intake of lavender essential oil (0.03 mL/kg), which contains a significant amount of LA, for 2 weeks did not have any disruptive effects on lipid profiles or liver enzymes in healthy athletes (Maral et al., 2022).

LA has been reported to have various therapeutic properties, including analgesic (Yu and Seol, 2017; Scuteri et al., 2022), antispasmodic (Rombolà et al., 2022), anti-psoriatic (Rai et al., 2020) and antibacterial (Mirzaei-Najafgholi et al., 2017; Ramić et al., 2021) effects. We have previously reported that LA is effective in preventing hypertension (Hsieh et al., 2018; Hsieh et al., 2019; Shin et al., 2022) and diabetes mellitus (Shin et al., 2018; Shin et al., 2020), which are risk factors for MCI. Moreover, LA has shown antioxidant and anti-inflammatory properties, as well as the ability to treat endothelial dysfunction (see Sections 3–5), suggesting that LA may have potential in preventing MCI. The present review briefly summarizes the roles of oxidative stress, inflammation, and endothelial dysfunction in MCI, as well as the benefits of LA in treating these MCI-associated factors. Additionally, because anxiety and mood disorders such as depression can affect cognition (Gulpers et al., 2019; Yuan et al., 2023), this review also considers the effects of LA on mood and cognition.

## 2 Oxidative stress, inflammation and endothelial dysfunction related to MCI

Epidemiological studies have suggested that hypertension and diabetes mellitus are risk factors for MCI. For example, cognitive performance was significantly lower in participants with uncontrolled hypertension than in other subjects (Lespinasse et al., 2022). In addition, the risk of MCI was significantly lower in hypertensive patients who did than did not effectively control their blood pressure (Yan et al., 2022). Mechanisms by which hypertension and diabetes mellitus can contribute to MCI include oxidative stress, inflammatory processes, and endothelial dysfunction (Ungvari et al., 2021). Hypertension has been found to increase NOX activity in vascular endothelial cells, thereby promoting ROS formation (Drummond and Sobey, 2014), and hyperglycemia due to diabetes mellitus stimulates ROS production via the advanced glycation end product, polyol, hexosamine, and protein kinase C pathways (Ighodaro, 2018). Increased oxidative stress can lead to MMP activity in brain tissue, which ultimately disrupts BBB integrity (Nath et al., 2019), and increased BBB permeability induces the activation of microglia (Ju et al., 2018). Activated microglia, in turn, produce inflammatory mediators, which further affect neuro-inflammation and neurodegeneration (Shabab et al., 2017).

**FIGURE 1:**
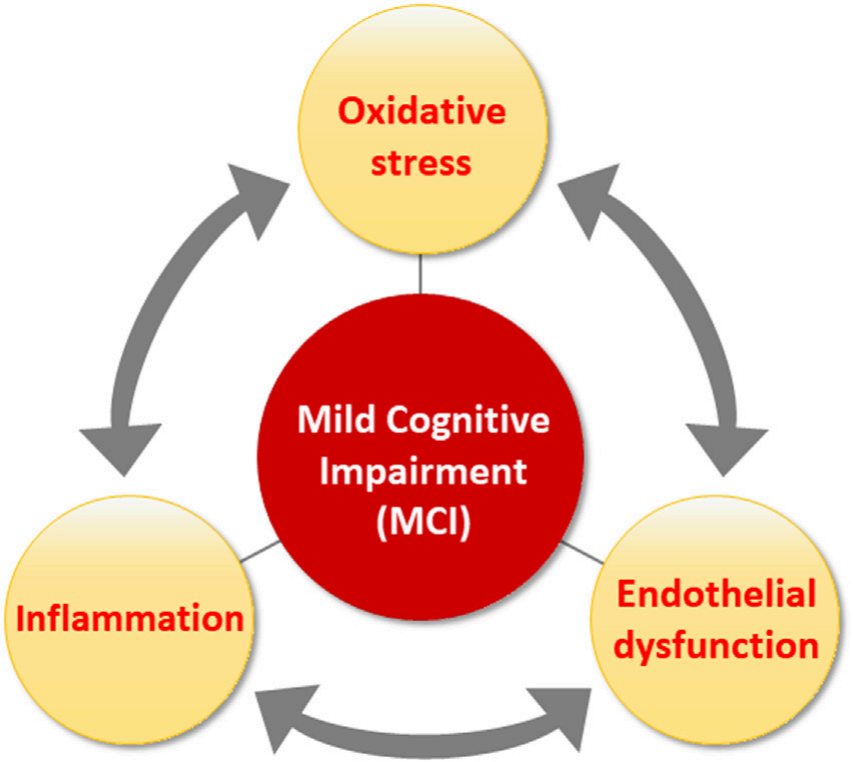
Overview of the relationships among oxidative stress, inflammation and endothelial dysfunction, factors involved in the pathology of MCI.

Oxidative stress can also interact with inflammatory responses, creating a vicious cycle that increases vascular endothelial dysfunction (Higashi, 2022). Oxidative stress due to an imbalance between ROS production and NO bioavailability results in endothelial dysfunction, ultimately leading to cardiovascular complications, including arterial stiffness (Burgos-Morón et al., 2019). Increased arterial stiffness contributes to the development of cerebrovascular dysfunction (Barnes and Corkery, 2018), which ultimately leads to cognitive impairment. In addition, inflammation mediated by ROS can increase BBB permeability, which increases the infiltration of immune cells, glial activation, and neuronal damage, further promoting neuro-inflammation (Van Dyken and Lacoste, 2018). Moreover, endothelial dysfunction can lead to endothelial activation (Liao, 2013). Proinflammatory cytokines, chemokines, and adhesion molecules are upregulated in activated endothelial cells, leading to inflammatory processes in blood vessels (Sun et al., 2020). Inflammation-induced oxidative stress increases ROS production in endothelial cells (Scioli et al., 2020). These findings indicate that oxidative stress and inflammation lead to endothelial dysfunction, which in turn enhances inflammatory responses and oxidative stress, forming a vicious cycle that may be associated with MCI (Figure 1).

## 3 Effects of LA on oxidative stress

Several *in vivo* and *in vitro* studies have shown that LA has antioxidant properties. Specifically, LA has been shown to have high peroxyl radical scavenger ability *in vitro* (Cutillas et al., 2018); to exert antioxidant effects in a rat model of combined hypertension and chronic obstructive pulmonary disease (COPD) by reducing MDA and lactate dehydrogenase (LDH) levels in serum (Hsieh et al., 2019); to inhibit cardiovascular disruption in rats treated with acute nicotine by restoring abnormally decreased heart rate and by reducing serum nitrite and LDH levels (Kim et al., 2017); to reduce systolic blood pressure in a rat model of hypertension-ischemia injury, a model that included the attenuation of p47^phox^ overexpression, ROS overproduction, and LDH release in the aorta (Hsieh et al., 2018); and to improve oxidative damage by reducing MDA levels in the liver tissue of diabetic rats (Shin et al., 2020). Moreover, under conditions mimicking Ca^2+^-related ischemic injury, LA was found to decrease NOX2 expression, ROS generation, and LDH release in microglial cells and to reduce p47^phox^ expression and LDH release in neuron-like cells, suggesting the protective roles of LA on the neurovascular unit (Hsieh et al., 2021).

Ca^2+^ is an intracellular second messenger that plays an important role in regulating cellular functions (Bootman and Bultynck, 2020). Agonist-induced Ca^2+^ entry into vascular endothelial cells induces nitric oxide (NO) production by endothelial nitric oxide synthase (eNOS) (Wei et al., 2018), and intracellular Ca^2+^ contributes to the contraction of vascular smooth muscle cells (Chen Y. L. et al., 2022). Neuronal processes, including the release of neurotransmitters and synaptic plasticity, are dependent on the fine-tuned regulation of intracellular Ca^2+^ levels (Brini et al., 2014). However, high concentrations of intracellular Ca^2+^ increase the activities of respiratory chain complexes, leading to excessive ROS formation, which further increases Ca^2+^ release from the endoplasmic reticulum (Görlach et al., 2015). LA has been reported to inhibit Ca^2+^ influx into human umbilical vein endothelial cells, indicating that it may have possible protective effects against endothelial dysfunction (You et al., 2013). Taken together, these findings demonstrating the antioxidant properties of LA suggest that LS has the potential to prevent MCI.

## 4 Effects of LA on inflammation

LA has also been found to have significant anti-inflammatory effects. LA has been shown to reduce skin levels of IL-1β and tumor necrosis factor (TNF)-α in mice with psoriasis-like skin lesions (Rai et al., 2020); to inhibit the activation of caspase-1 and nuclear factor-κB (NF-κB) in a human mast cell line exposed to inflammatory stimuli (Moon et al., 2018); to inhibit the expression of cell adhesion molecules and NF-κB activation in murine brain endothelial cells stimulated with TNF-α (Aoe et al., 2017); and to exert anti-inflammatory effects in a rat model of combined COPD and hypertension by reducing TNF-α, IL-6, and MMP-9 levels in bronchoalveolar lavage fluid (Hsieh et al., 2019). Moreover, LA was effective in reducing systolic and diastolic blood pressure in rats with repeatedly stressed-ulcerative colitis, as well as reducing serum IL-6 concentrations (Shin et al., 2022). LA also significantly reduced serum glucose levels in rats with streptozotocin-induced diabetes exposed to chronic immobilization stress, which resulted from increased liver AMP-activated protein kinase expression, decreased liver NF-κB expression, and excessive amounts of serum nitrite (Shin et al., 2018).

A sustained increase in intracellular Ca^2+^ levels has been associated with the activation of microglia (Brawek and Garaschuk, 2013), further emphasizing the importance of maintaining intracellular Ca^2+^ homeostasis. In this regard, LA has been shown to decrease store-operated Ca^2+^ entry elevation in microglial cells under the conditions of muscarinic receptor blockade and inflammatory stimulus (Kim et al., 2022).

Skeletal muscle releases several myokines, including insulin-like growth factor (IGF)-1, which have pleiotropic effects. IGF-1 is responsible for maintaining skeletal muscle mass (Bian et al., 2020), and has been shown to have anti-inflammatory and antioxidant properties (Sukhanov et al., 2007). IGF-1 deficient mice exhibited significantly reduced expression of Nrf2 in the aorta, which makes the vasculature susceptible to oxidative stress (Bailey-Downs et al., 2012). Also, mice with IGF-1 deficiency showed increased *Tnfa* and *Il1b* mRNAs in the retina indicating persistent inflammation (Arroba et al., 2016). In addition, activation of the IGF-1/PI3K/AKT/GSK-3β pathway was shown to attenuate neuro-inflammation and cognitive impairment after sleep deprivation (Wan et al., 2022). LA was shown to prevent losses in body weight and gastrocnemius muscle weight in rats with rheumatoid arthritis exposed to chronic nicotine treatment. Mechanistically, LA increased muscle fiber cross-sectional area and serum IGF-1 levels in these rats and decreased serum IL-6 levels and mitochondrial membrane potential in the gastrocnemius muscle. Moreover, LA was more potent than lavender essential oil in enhancing serum IGF-1 concentrations (Seo et al., 2021). These findings indicate that LA is an effective anti-inflammatory agent, which may attenuate the cascade of events leading to MCI.

## 5 Effects of LA on endothelial dysfunction

LA has also been shown to reduce endothelial dysfunction. For example, LA reduced ROS-induced eNOS suppression in the aorta in a rat model of hypertension-ischemia injury (Hsieh et al., 2018); improved endothelial function by increasing eNOS expression and acetylcholine (ACh)-induced vasorelaxation in the aortas of diabetic rats (Shin et al., 2018); and increased NO and decreased MMP-9 expression, a key marker of BBB disruption, under conditions mimicking Ca^2+^-related ischemic injury in a mouse brain endothelial cell line (Hsieh et al., 2021). Taken together, these findings suggest that LA may prevent MCI associated with muscle wasting. Overall, the findings presented in Sections 3–5 suggest that LA has the potential to prevent the development of MCI by reducing oxidative stress, inflammation, and endothelial dysfunction (Table 1).

**TABLE 1:** Summary of the effects of LA on oxidative stress, inflammation and endothelial dysfunction.

Models	Doses	Effects	Mechanisms	References
SD rats, male, Acute nicotine exposure	1, 10 or 100 mg/kg	Inhibiting cardiovascular disruption	Serum nitrite, LDH↓	Kim et al. (2017)
SD rats, male, Repeatedly stressed-ulcerative colitis	10 or 100 mg/kg	Anti-hypertensive; Anti-inflammatory	Systolic BP, diastolic BP↓	Shin et al. (2022)
			Colon nitrite↓	
			Serum IL-6 (decreasing tendency)	
SD rats, male, Hypertensive ischemic injury	25, 50 or 100 mg/kg	Anti-hypertensive; Anti-oxidant	Systolic BP↓; Aorta eNOS↑	Hsieh et al. (2018)
			Aorta p47^phox^, ROS, LDH↓	
SD rats, male, COPD-like and hypertension	1, 10 or 100 mg/kg	Anti-hypertensive; Anti-oxidant; Anti-inflammatory	Systolic BP↓	Hsieh et al. (2019)
			Lung NF-κB↓	
			BAL fluid TNF-α, IL-6, MMP-9↓	
			Serum MDA, LDH↓	
			Serum DPPH (increasing tendency)	
EA.hy926 cells	0.01%	Intracellular Ca^2+^ homeostasis	Intracellular Ca^2+^ concentration (↑transiently)	You et al. (2013)
			Ca^2+^ influx↓	
SD rats, male, Chronic stress and STZ-induced DM	10 or 100 mg/kg	Anti-diabetic; Enhancing endothelium-dependent vasorelaxation; Anti-inflammatory	Blood sugar↓, Liver AMPK↑	Shin et al. (2018)
			Abdominal artery eNOS↑	
			ACh-induced vasorelaxation↑	
			Liver NF-κB↓	
SD rats, male, Chronic stress, high-fat diet and STZ-induced DM	1 or 10 mg/kg	Anti-diabetic; Anti-stress; Anti-oxidant; Anti-inflammatory	Fasting blood sugar, HOMA-IR↓	Shin et al. (2020)
			Serum insulin levels↓	
			Serum corticosterone↓	
			Liver mitochondrial membrane potential↑	
			Pancreas NF-κB and liver MDA (decreasing tendency)	
SD rats, male, Collagen-induced arthritis exposed to chronic nicotine	100 mg/kg	Inhibiting muscle wasting; Anti-inflammatory	Gastrocnemius muscle weight↑	Seo et al. (2021)
			Hind paw thickness↓	
			Muscle fiber cross-sectional area↑	
			Gastrocnemius muscle mitochondrial membrane potential↓	
			Serum IL-6↓, Serum IGF-1↑	
bEnd.3, SH-SY5Y, BV2, and U373 cells, Ca^2+^-related ischemic injury	500 μM	Anti-oxidant Protecting BBB	bEnd.3: NO↑, MMP-9, LDH↓	Hsieh et al. (2021)
			SH-SY5Y: p47^phox^, LDH↓	
			BV2: NOX2, ROS, LDH↓	
			U373: ONOO^−^, p47^phox^↓	
SH-SY5Y and BV2 cells, Inflammatory stimulus and muscarinic receptor blockade	500 μM	Intracellular Ca^2+^ homeostasis	Decreasing SOCE by activating the forward mode of NCX and the Na^+^/K^+^ ATPase	Kim et al. (2022)
BALB/c mice, female, Imiquimod-induced psoriasis-like skin lesion	1% or 2%	Anti-inflammatory	Skin IL-1β, TNF-α↓	Rai et al. (2020)
HMC-1 cells, Phorbol myristate acetate plus A23187 stimulation	400 μg/mL	Anti-inflammatory	Caspase-1, NF-κB, TSLP↓ Ca^2+^ influx↓	Moon et al. (2018)
bEnd.3 cells, TNF-α stimulation	62.5 or 125 μM	Anti-inflammatory	E-selectin, P-selectin↓ VCAM-1, ICAM-1, NF-κB↓	Aoe et al. (2017)

Abbreviations: AMPK, AMP-activated protein kinase; ACh, acetylcholine; BAL, bronchoalveolar lavage; BP, blood pressure; COPD, chronic obstructive pulmonary disease; DM, diabetes mellitus; DPPH, 2,2-Diphenyl-1-picrylhydrazyl; eNOS, endothelial nitric oxide synthase; HOMA-IR, homeostatic model assessment-insulin resistance; ICAM, intercellular adhesion molecule; IGF, insulin-like growth factor; IL, interleukin; LDH, lactate dehydrogenase; MDA, malondialdehyde; MMP, matrix metalloproteinase; NCX, Na^+^/Ca^2+^ exchanger; NF-κB, nuclear factor-κB; NO, nitric oxide; NOX, NADPH, oxidase; ONOO^−^, peroxynitrite; ROS, reactive oxygen species; SD, sprague dawley; SOCE, store-operated Ca^2+^ entry; STZ, streptozotocin; TNF, tumor necrosis factor; TSLP, thymic stromal lymphopoietin; VCAM, vascular cell adhesion molecule.

## 6 Effects of LA on mood and cognition

Anxiety and depression are common in individuals with MCI, thereby increasing the risk of progression to dementia (Ma, 2020). Moreover, psychological distress and depression have been identified as longitudinal predictors of cognitive decline in older adults (Freire et al., 2017).

LA has been shown to have positive effects on mood. Inhalation of LA by cancer patients prior to chemotherapy decreased the anxiety-visual analogue scale (VAS) and the stress-VAS scores in cancer patients, suggesting that LA has anti-anxiety and anti-stress effects (Kim et al., 2021). Inhalation of lavender, petitgrain, or bergamot essential oil containing large amounts of LA was found to reduce the Anger-Hostility and the Tension-Anxiety scores in pregnant women (Igarashi, 2013). Lavender essential oil showed anxiolytic effects in mice by increasing the time spent in the open arm of the elevated plus maze test (Schuwald et al., 2013). Clary sage essential oil containing LA as its major bioactive component showed an antidepressant-like effect by reducing immobility time in the forced swimming test, an effect blocked by a dopamine antagonist (Seol et al., 2010). A study using bioinformatics tools found that LA appeared to modulate serotonin transporters and to have a strong affinity to serotonin 1A and dopamine D_2_ receptors, indicating that LA may be promising as an antidepressant (Avram et al., 2021). In addition, the abilities of LA to increase parasympathetic activity (Igarashi, 2013), inhibit Ca^2+^ influx (Schuwald et al., 2013), and inhibit sedative activity (Buchbauer et al., 1991) may be related to its mood-enhancing effects.

Studies have reported that LA may play a role in cognitive function. Because proper Ca^2+^ signaling is important in regulating neuronal function, such as synaptic plasticity, abnormal Ca^2+^ signaling can lead to synaptic loss in neurodegenerative diseases (Pchitskaya et al., 2018). Exposure of mice to lipopolysaccharide (LPS) decreased learning and memory functions, through mechanisms associated with increased ROS production and NOX2 expression in brain tissue, as well as neuronal inflammation (Dong et al., 2021). Moreover, exposure to LPS significantly increased intracellular Ca^2+^ overload in a murine hippocampal cell line showing Ca^2+^ dyshomeostasis (Dong et al., 2021). LA also reduced store-operated Ca^2+^ entry elevation induced by muscarinic receptor inhibition and inflammatory stimuli by activating the forward mode of Na^+^/Ca^2+^ exchanger and Na^+^/K^+^ pump in neuron-like and microglial cells (Kim et al., 2022). Under conditions mimicking Ca^2+^-related ischemic injury, LA decreased p47^phox^ expression and LDH release in neuron-like cells and reduced NOX2 expression, ROS generation, and LDH release in microglial cells (Hsieh et al., 2021). These findings indicate that LA can protect neurons and microglia against oxidative stress and inflammatory responses.

Acetylcholine is a cholinergic neurotransmitter that plays an important role in cognitive function (Chen Z. R. et al., 2022). However, acetylcholinesterase (AChE), an enzyme whose primary function is to degrade acetylcholine, inhibits postsynaptic signal transmission (Singh and Gupta, 2017). In the absence of AChE, butyrylcholinesterase (BChE) can compensate, by hydrolyzing acetylcholine (Mesulam et al., 2002). LA has been reported to have AChE and BChE inhibitory activities, with IC_50_ values of 82 μg/mL (Miyazawa et al., 1998) and 169 μg/mL (Bonesi et al., 2010), respectively. Molecular docking simulations also showed that LA had a high affinity for the binding site of BChE (Lobine et al., 2021), further suggesting that LA may be useful for improving cognitive function in subjects with MCI.

## 7 Overall effects of LA against MCI-associated factors

**FIGURE 2:**
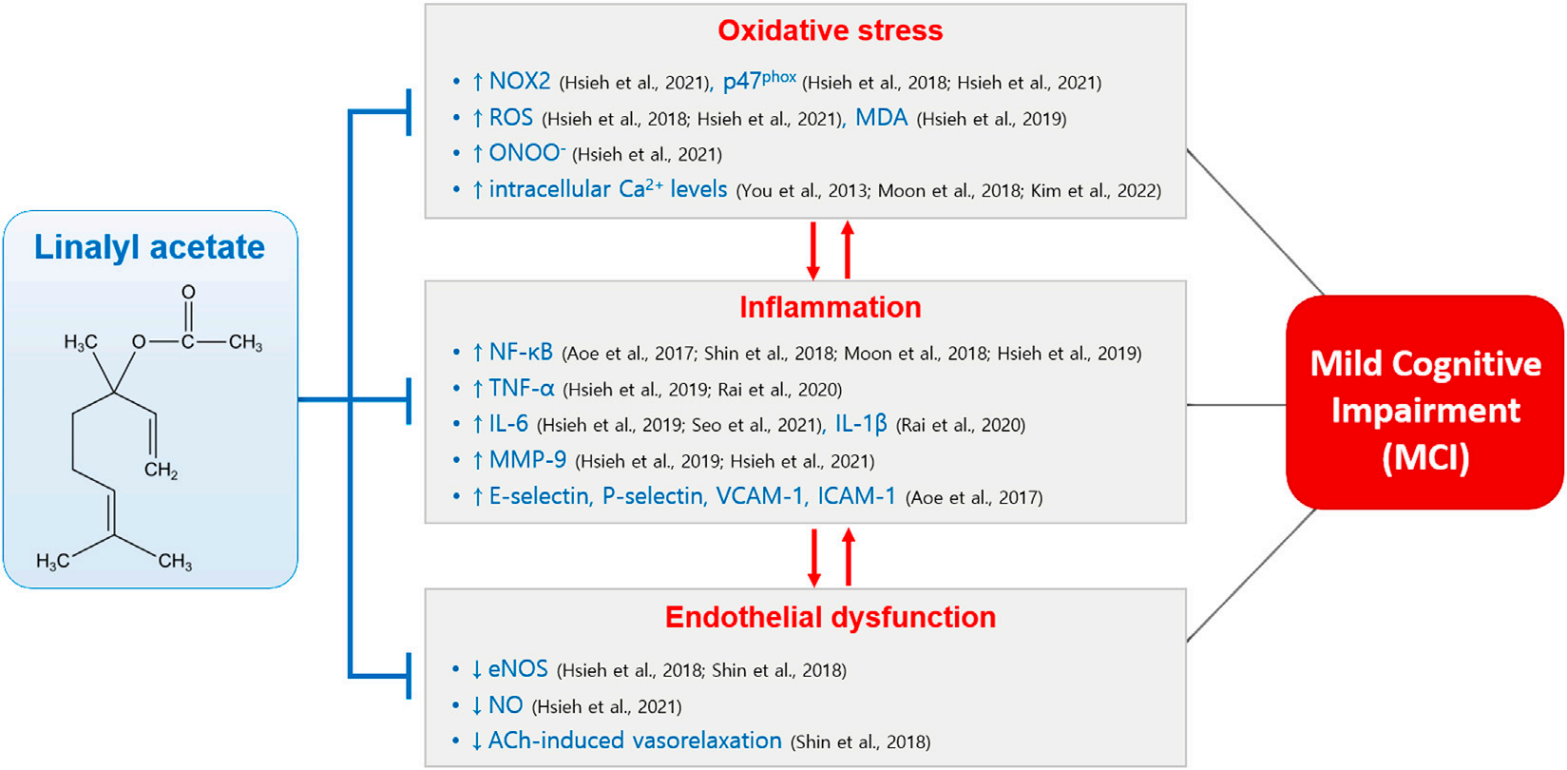
Possible methods of action of LA in preventing MCI by reducing oxidative stress, inflammation and endothelial dysfunction. Abbreviations: ACh, acetylcholine; eNOS, endothelial nitric oxide synthase; ICAM, intercellular adhesion molecule; IL, interleukin; MDA, malondialdehyde; MMP, matrix metalloproteinase; NF-κB, nuclear factor-κB; NO, nitric oxide; NOX, NADPH oxidase; ONOO^−^, peroxynitrite; ROS, reactive oxygen species; TNF, tumor necrosis factor; VCAM, vascular cell adhesion molecule.

In summary, LA has proved to have antioxidant effect by reducing NOX2 (Hsieh et al., 2021), p47^phox^ (Hsieh et al., 2018; Hsieh et al., 2021), ROS (Hsieh et al., 2018; Hsieh et al., 2021), MDA (Hsieh et al., 2019), peroxynitrite (ONOO^−^) (Hsieh et al., 2021), and intracellular Ca^2+^ levels (You et al., 2013; Moon et al., 2018; Kim et al., 2022). Also, LA has been reported to have anti-inflammatory activity by reducing NF-κB activation (Aoe et al., 2017; Moon et al., 2018; Shin et al., 2018; Hsieh et al., 2019), TNF-α production (Hsieh et al., 2019; Rai et al., 2020), IL-6 production (Hsieh et al., 2019; Seo et al., 2021), IL-1β production (Rai et al., 2020), MMP-9 production (Hsieh et al., 2019; Hsieh et al., 2021), and E-selectin, P-selectin, vascular cell adhesion molecule-1, and intercellular adhesion molecule-1 expression (Aoe et al., 2017). In addition, it has been reported that LA has the ability to reverse endothelial dysfunction by increasing eNOS expression (Hsieh et al., 2018; Shin et al., 2018), NO production (Hsieh et al., 2021), and ACh-induced vasorelaxation (Shin et al., 2018). Therefore, LA may prevent the development of MCI by mitigating MCI-associated factors such as oxidative stress, inflammation, and endothelial dysfunction (Figure 2). Additionally, LA is effective in reducing anxiety and depressive symptoms related to cognitive decline.

## 8 Conclusion and future perspectives

In conclusion, numerous *in vivo* and *in vitro* studies have shown that LA is an effective antioxidant and anti-inflammatory agent, and that it reduces endothelial dysfunction, suggesting that LA can prevent the development of MCI. Importantly, LA does not have genotoxic, phototoxic, photoallergenic, or skin-sensitization properties (Api et al., 2015). However, efforts to develop LA as a drug molecule must overcome some challenges. Main issues are that the pharmacokinetic and pharmacodynamic properties of LA are insufficient for clinical applications. For example, although LA penetrated through the skin of healthy male subjects after massage with LA-containing lavender essential oil, the maximum concentration of LA in blood was only 100 ng/mL, which is insufficient to have a clinical effect (Jäger et al., 1992). In addition, the blood levels of LA in mice that inhaled LA at a concentration of 5 mg/L air were found to be only 1–2 ng/mL (Jirovetz et al., 1991). Moreover, when LA is taken orally, the compound is metabolized to linalool and α-terpineol by carboxylesterase in gastric juice, following which both linalool and α-terpineol are conjugated and oxidized to more polar metabolites and excreted (Bickers et al., 2003). Another problem is the low solubility of LA in water (0.054 mg/mL) (Cal, 2006). Additional studies, therefore, are needed to investigate the pharmacokinetic and pharmacodynamic properties of LA, and to verify the effects of LA in patients at risk of developing MCI. Collectively, the results summarized in the present review provide a basis for the development of new strategies for preventing MCI using LA.
